# First Case Report on Quantification of Antimicrobial Use in Corporate Dairy Farms in Pakistan

**DOI:** 10.3389/fvets.2020.575848

**Published:** 2020-11-26

**Authors:** Muhammad Umair, Rana Muhammad Abdullah, Bilal Aslam, Muhammad Hassan Nawaz, Qasim Ali, Fariha Fatima, Jabir Ali, Muhammad Asif Zahoor, Mashkoor Mohsin

**Affiliations:** ^1^Institute of Microbiology, University of Agriculture, Faisalabad, Pakistan; ^2^Institute of Pharmacy, Physiology and Pharmacology, University of Agriculture, Faisalabad, Pakistan; ^3^Department of Theriogenology, Faculty of Veterinary Science, University of Agriculture, Faisalabad, Pakistan; ^4^Department of Microbiology, Faculty of Life Sciences, Government College University Faisalabad, Faisalabad, Pakistan

**Keywords:** antimicrobial use, quantification, LMICs, corporate dairy, Pakistan

## Abstract

Intensive livestock farming has become indispensable to meet the rapidly increasing demand for animal-based nutrition in low- and middle-income countries (LMICs) where antimicrobials are frequently used for treatment and prophylactic or metaphylactic purposes. However, very little is known about the trends of antimicrobial use (AMU) in dairy animals in LMICs. The objective of this study was to quantify AMU in two large commercial dairy farms in Pakistan. A retrospective study was conducted at two large corporate commercial dairy farms located in Punjab province for the year 2018. AMU was calculated using three metrics: active ingredient (AI; kg) and milligrams per population unit (mg/PU; mg/kg), which quantifies the amount of AI used, and antimicrobial treatment incidence (ATI; DDDA/1,000 cow-days), which estimates the per-day number of treatments to 1,000 cows. Total on-farm AMU was found to be 138.34 kg, 65.88 mg/kg, and 47.71 DDDA/1,000 cow-days. Measured in ATI, aminoglycosides (11.05 DDDA/1,000 cow-days), penicillins (8.29 DDDA/1,000 cow-days), and tetracyclines (8.1 DDDA/1,000 cow-days) were the most frequently used antimicrobial classes. A total of 42.46% of all the antimicrobials used belonged to the critically important antimicrobials for human medicine as defined by the World Health Organization. Considerably high AMU was found compared to other farm-level studies across the world. This was the first study to quantify AMU in the dairy industry in Pakistan. Our results showed that corporate commercial dairy management practices are associated with increased antimicrobial consumption and highlight the need for antimicrobial stewardship programs to encourage prudent use of antimicrobials in commercial dairy.

## Introduction

Antimicrobial resistance (AMR) has been considered a global health problem, and the situation is worse in low- and middle-income countries (LMICs) due to lack of responsible antimicrobial use (AMU) and inadequate antimicrobial stewardship (1). Owing to the increasing demand of animal protein, antimicrobials are extensively used in food animals for treatment and prophylactic reasons (2, 3). Global data highlighted that the overall consumption of antimicrobials in food animals far exceeds consumption in human medicine because of larger biomass and non-therapeutic AMU in food animals (4–6). It has been suggested that the overall burden of AMR has increased due to the contribution of AMU from food animals (7). In addition, AMU in animals has become a worldwide concern as in most of the countries, more than 50% of the medically important antimicrobials are being used in livestock (5). In efforts to meet the ever-increasing animal protein demand in LMICs, a shift towards intensive livestock farming has resulted in irrational AMU (3, 8). However, data on AMU in LMICs are often not available due to weak regulatory infrastructure, over-the-counter sale of antimicrobials, and inappropriate prescription practices (9). Surveillance of AMU in animal production systems is one of the key objectives of the Global Action Plan on AMR (GAP-AMR) proposed by the World Health Organization (WHO) in the 68th World Health Assembly in 2015 (10). As a WHO member state, Pakistan has drafted its National Action Plan on AMR (NAP-AMR) in 2017 (11).

The dairy sector in Pakistan plays a significant role in its agriculture-based economy. Pakistan is one of the world's top milk producers, with an estimate of 45.8 million tons of milk produced in the year 2018 (12). The majority of dairy milk is produced from small dairy holders (one to four animals) throughout the country; however, due to the increasing demand of milk intensive and semi-intensive dairy farming is becoming increasingly popular (13, 14).

According to FAOSTAT 2018, Pakistan stands at 3rd (13.6 million cattle heads) and 11th (16.8 million tons) in terms of the number of cattle and per-year milk production, respectively, but 128th (3.4 L/day) in terms of yield per animal (12). Livestock in Pakistan contributes to 60.54% in the agriculture sector and 11.22% in the country's GDP with a growth of 4% during the fiscal year 2018–2019. The livestock wing under the Ministry of National Food Security and Research has taken several measures for the growth of the dairy sector in terms of improving per-unit productivity by allowing the import of high-yielding exotic dairy breeds (Holstein-Friesian and Jersey), their genetic material (embryos and semen), feedstuff, and farm equipment at low import duties. Dairy production in Pakistan can be classified into five major systems, i.e., small holder subsistence or market-oriented production system, rural or peri-urban commercial production system, and corporate sector production system. The corporate sector represents <1% of the country's dairy and maintains high-producing exotic cattle breeds (Holstein-Friesian and Jersey) with an average herd size of 2,000–5,000 animals. Currently, only 15 such large corporate farms are operating in the country (14, 15).

Although NAP-AMR urged the monitoring and reduction in the level of AMU in animals, nationwide surveillance to monitor AMU in livestock has not been established yet (16). Therefore, this study is designed to quantify AMU on a convenience sample of two large corporate commercial dairy farms for 1 year to provide the first baseline study on AMU at the farm-level in Pakistan.

## Materials and Methods

### Study Design

To evaluate quantitative AMU, a retrospective study was conducted in two large corporate commercial dairy farms located in Punjab province for the year 2018. Both farms were automated semi-controlled and had maintained exotic cattle (Holstein-Friesian) with a total animal count of 5,588, consisting of 2,480 milking cows, 1,506 heifers, and 1,602 calves, taken as average for the year 2018 (Table 1). As a standard commercial operation, inventory records were maintained at both farms.

**Table 1:** Adjusted animal number (ANadj) as per the weights of dairy cattle defined by Jensen et al. (17).

**Category**	**Count^**a**^**	**Weight/Head (kg)**	**ANadj**	**Biomass (kg)**
Farm 1				
Cows	624	600	624	374,400
Heifers	576	300	288	172,800
Calves	528	100	88	52,800
Farm 2				
Cows	1,856	600	1,856	1,113,600
Heifers	930	300	465	279,000
Calves	1,074	100	179	107,400
Total	5,588		3,500	2,100,000^b^

a *Animal count taken as year average*.

b *Population unit (PU); composite weight (Wc) of all the animals under study*.

Drug inventory output records (drugs issued from inventory intended to be used at animals kept on the farm) from January 1st to December 31st for the year 2018 were accessed after signing an agreement permitting drug inventory data access and ensuring farm anonymity and secrecy in any form of publication. For the detailed product composition and dosage information, market, and respective product websites were visited. Data were maintained in columns as product name, active ingredients (AIs), concentration, and labeled dosage. Calculations were made for each AI, categorized regarding its class and the labeled treatment route using Microsoft Office Excel 2016. AMU was calculated in three different metrics: 1) AI, 2) antimicrobial treatment incidence (ATI), and 3) milligrams per population unit (mg/PU). Metrics 1 and 3 quantify the amount of AI used, whereas metric 2 estimates the number of treatments per day over 1,000 cows.

### Active Ingredient

The total amount of AI in milligrams (mg) for each product is determined using the labeled concentration and quantity used. For products comprising more than one AI, mg were calculated separately for each ingredient. For the prodrug compositions and the concentrations given in international units (IU), AI mg were calculated using the methodology defined by World Organization for Animal Health (OIE) and European Surveillance of Veterinary Antimicrobial Consumption (ESVAC) [European Medicine Agency (EMA)] (18, 19). For each antimicrobial, AI values were then added up and expressed in kilograms (kg).

### Antimicrobial Treatment Incidence

Total amount of AI used in terms of the number of treatments as per the defined daily dose animal (DDDA) over 1,000 cows per day is evaluated as ATI (**Equations 1 and 2**). One DDDA (mg/cow-day) is defined as the average labeled daily dose, recommended to be administered per day, in mg per kg of the animal body weight multiplied by the approximate body weight of a dairy cow taken as 600 kg (**Equation 3**) (17). For long-acting compositions, DDDA was calculated as per-day average according to the labeled duration of action (20). For products containing more than one AI, DDDA for each AI were calculated according to the labeled daily dose mentioned for the respective product. For intramammary compositions, one intramammary tube was considered as one DDDA (21, 22). As the drug inventory records at both farms were maintained for the entire on-farm herd population, the total animal biomass is adjusted against the weight of one dairy cow (23), and the total number of animals was calculated to be 3,500 termed as adjusted animal number (ANadj) (Table 1) (**Equation 4**). ANadj is used for the calculation of ATI for each AI against every product in the drug inventory (ATI for all AIs in a single product will be the same) (**Equations 1**–**4**). Individual ATIs were then added up for each antimicrobial and drug class with respect to the labeled route of treatment.

(1) $$\begin{array}{l} ATI_{DDDA/1,000cow-days}=TF\times 1,000_{cow-days} \end{array}$$

(2) $$\begin{array}{l} TF=\frac{{Total\;amount\;of\;individual\;AI\;for\;each\;brand\;used}_{mg}}{DDDA_{mg/cow-day}\;\times ANadj_{\;cows}\times {Days\;of\;study}_{days}} \end{array}$$

(3) $$\begin{array}{l} DDDA_{mg/cow-day}={Labeled\;daily\;dose}_{mg/kg}\times \;600_{kg} \end{array}$$

(4) $$\begin{array}{l} ANadj=\frac{Total\;biomass\;\left(Wc_{kg}\right)}{600_{kg}} \end{array}$$

Treatment fraction (TF) is a decimal ratio between the actual numbers of treatments and the maximum possible number of treatments within the days of study, i.e., *ANadj* × *Days of study*, also termed as animal-days at risk. TF when multiplied by 1,000 cow-days gives the number of treatments using DDDA per thousand cows in 1 day, i.e., DDA/1,000 cow-days.

### Milligrams per Population Unit

Population unit (PU) is defined as the composite weight (Wc) in kg of all the animals in the study. mg/PU is the amount of AI in mg used per kg of PU. PU was considered constant throughout the study period.

(5) $$\begin{array}{l} {mg/PU}_{mg/kg}=\frac{AI_{mg}}{Wc_{kg}} \end{array}$$

## Results

A total of 42 antimicrobial products (parenteral, intramammary, and intrauterine) were used, containing 28 different AIs (belonging to 13 antimicrobial classes) during the year 2018 (Tables 2, 3, Supplementary Table 1). Of the 42 antimicrobial products used, 15 contained a single antimicrobial agent (five each, belonging to aminoglycosides, cephalosporins, and tetracyclines), whereas 27 were combination products (Table 3). In terms of ATI metric, aminoglycosides (11.05 DDDA/1,000 cow-days), penicillins (8.29 DDDA/1,000 cow-days), and tetracyclines (8.1 DDDA/1,000 cow-days) were the most frequently used antimicrobial classes, whereas sulfonamides (34.16 kg, 16.27 mg/kg), aminoglycosides (33.17 kg, 15.79 mg/kg), and tetracyclines (31.42 kg, 14.96 mg/kg) were the most highly used antimicrobial classes when measured in quantities as AI and mg/PU. Parenterally administered antimicrobials gave the highest total ATI, i.e., 23.49 DDDA/1,000 cow-days followed by intramammary, oral, and intrauterine routes with a total ATI of 21.07, 3.05, and 0.1 DDDA/1,000 cow-days, respectively (Table 2).

**Table 2:** Active ingredient (AI), treatment fraction (TF), antimicrobial treatment incidence (ATI), and milligrams of active ingredient used per kilogram of total population weight (mg/PU) of different antimicrobial classes in the study.

**Antimicrobial** **class**	**Parenteral**			**Intramammary**			**Intrauterine**			**Oral**			**Total**			
	**AI^**a**^**	**TF^**b**^**	**ATI^**c**^**	**AI**	**TF**	**ATI**	**AI**	**TF**	**ATI**	**AI**	**TF**	**ATI**	**AI**	**TF**	**ATI**	**mg/PU^**d**^**
Aminocoumarins	–	–	–	0.17	0.0013	1.32	–	–	–	–	–	–	0.17	0.0013	1.32	0.08
Aminoglycosides	31.55	0.0044	4.43	0.91	0.0056	5.57	0.01	0	0.03	0.7	0.001	1.02	33.17	0.0111	11.05	15.79
Aminopenicillins	4.72	0.0007	0.73	0.18	0.0019	1.88	–	–	–	–	–	–	4.9	0.0026	2.61	2.33
Aminopenicillins+β-lactam inhibitors	0.0088	–	0.0013	–	–	–	–	–	–	–	–	–	0.0088	–	0.0013	0.0042
Antifungals	0.0009	–	0.03	–	–	–	–	–	–	–	–	–	0.0009	–	0.03	0.0004
Cephalosporins	0.73	0.001	0.96	1.52	0.0049	4.9	–	–	–	–	–	–	2.25	0.0059	5.85	1.07
Fluoroquinolones	6.9	0.0037	3.72	–	–	–	–	–	–	–	–	–	6.9	0.0037	3.72	3.29
Macrolides	3.96	0.0015	1.47	–	–	–	–	–	–	–	–	–	3.96	0.0015	1.47	1.89
Penicillins	8.11	0.003	3	0.84	0.0053	5.25	0.01	–	0.03	–	–	–	8.96	0.0083	8.29	4.27
Phenicols	11.75	0.0012	1.18	–	–	–	–	–	–	–	–	–	11.75	0.0012	1.18	5.6
Polymyxins	0.65	0.0009	0.86	–	–	–	–	–	–	–	–	–	0.65	0.0009	0.86	0.31
Polypeptides	–	–	–	0.04	0.0011	1.08	–	–	–	–	–	–	0.04	0.0011	1.08	0.02
Sulfonamides	8.92	0.0001	0.08	–	–	–	0.35	–	0.03	24.89	0.002	2.03	34.16	0.0022	2.15	16.27
Tetracyclines	31.15	0.007	7.02	0.28	0.0011	1.08	–	–	–	–	–	–	31.42	0.0081	8.1	14.96
Total	108.45	0.0235	23.49	3.93	0.0211	21.07	0.37	0.0001	0.1	25.59	0.0031	3.05	138.34	0.0477	47.71	65.88

a *AI: the amount of active ingredient used in kilogram*.

b *TF: a ratio between the actual numbers of treatments and the maximum possible number of treatments and has no unit. TF values are rounded to four digits after decimal; complete values are given in Supplementary Table 1*.

c *ATI: the number of antimicrobial treatments per 1,000 cow–days in DDDA/1,000 cow-days*.

d *mg/PU: milligrams of active ingredient used per kilogram of total animal biomass in milligrams per kilogram*.

**Table 3:** Formulations of 42 products used at the studied farms.

	**AMG**	**AMG**	**AMG**	**AMG**	**AMG**	**AMP**	**CEP**	**FLQ**	**MAC**	**MAC**	**PEN**	**PEN**	**PHN**	**SUL**	**SUL**	**TET**	
	5					2	5	3			1		3	1		5	
**AMM**		1^a^															**PEN**
**AMG**						1	1		1	1	1	1					
**AMP**											1						
**MAC**													1				
**PEN**			2	1											1		
**POP**																	
**SUL**															2		
**TET**					1												
**β-L**						1											
		**AMG**	**PEN**	**ANF**	**POP**					**POM**		**PEN**			**AMG**		

a *Table key: In one product containing four active ingredients, classes are mentioned in order of decreasing concentration (mg/ml), i.e., AMG, AMM, AMG, and PEN. Numbers at the intersection of more than one antimicrobial class represent products with more than one antimicrobial active ingredient*.

*AMG, Aminoglycosides; AMP, Aminopenicillins; CEP, Cephalosporins; FLQ, Fluoroquinolones; MAC, Macrolides; PEN, Penicillins; PHN, Phenicols; SUL, Sulfonamides; TET, Tetracyclines; AMM, Aminocoumarins; POP, Polypeptides; β-L, β-Lactams; ANF, Antifungals; POM, Polymyxins*.

AI analysis showed that in terms of ATI, oxytetracycline (7.02 DDDA/1,000 cow-days), penicillin G (6.24 DDDA/1,000 cow-days), and cefalonium (4.27 DDDA/1,000 cow-days) were the most frequently used antimicrobial AIs. Oxytetracycline (7.02 DDDA/1,000 cow-days), gentamicin (3.34 DDDA/1,000 cow-days), and enrofloxacin (3.11 DDDA/1,000 cow-days) were the most frequent antimicrobials used via parenteral administration. ATI values for the antimicrobials cefalonium (4.27 DDDA/1,000 cow-days), penicillin G (3.21 DDDA/1,000 cow-days), and neomycin (2.56 DDDA/1,000 cow-days) used in intramammary compositions were higher than those for the parenteral compositions, i.e. 0, 3, and 0 DDDA/1,000 cow-days, respectively. In terms of the AI quantities, oxytetracycline (31.15 kg, 14.83 mg/kg), streptomycin (21.35 kg, 10.17 mg/kg), and sulfadimidine (19.99 kg, 9.52 mg/kg) were commonly used antimicrobials (Supplementary Table 1). A total of 138.34 kg of antimicrobials was used at the studied farms during the year 2018 with ATI and mg/PU of 47.71 DDDA/1,000 cow-days and 65.88 mg/kg, respectively (Table 2, Supplementary Table 1).

A total of 21% (6/28) of the antimicrobials used at studied farms belonged to the critically important with highest priority (CIA-HtP) category of antimicrobials for human medicine, while 32% (9/28) belonged to the high priority (CIA-HhP) category according to WHO (Table 4) (24). ATI quantities for CIA-HtP and CIA-HhP were 7.03 and 19.91 DDDA/1,000 cow-days, whereas quantities used were 12.24 and 46.49 kg, respectively (Table 4).

**Table 4:** Total active ingredient (AI) kilogram (kg) and percentage (%), antimicrobial treatment incidence (ATI), and milligrams of active ingredient used per kilogram of total population weight (mg/PU) by WHO Critically Important Antimicrobial (CIA) for Human Medicine, sixth revision (24).

**WHO CIA Category**	**Antimicrobials used^**a**^**	**AI (kg)**	**AI (%)**	**ATI (DDDA/1,000** **cow-days)**	**mg/PU** **(mg/kg)**
Critically Important Antimicrobials with Highest Priority (CIA-HtP)	Ceftiofur, cefquinome, tylosin, colistin, enrofloxacin, marbofloxacin	12.24	8.85	7.03	5.83
Critically Important Antimicrobials with High Priority (CIA-HhP)	Dihydrostreptomycin, framycetin, gentamicin, neomycin, streptomycin, amoxicillin, ampicillin, clavulanic acid, penicillin G	46.49	33.61	19.91	22.14
Highly Important Antimicrobials (HIA)	Cefalonium, cephalexin, cloxacillin, oxytetracycline, tetracycline, sulphadiazine, sulfadimidine, sulfathiazole	67.65	48.9	17.16	32.21
Important Antimicrobials (IA)	Bacitracin, florfenicol, thiamphenicol	11.79	8.52	2.26	5.61
Others	Novobiocin, methyl hydroxybenzoate	0.17	0.12	1.35	0.08

a *Supplementary Table 1*.

## Discussion

Globally, several initiatives have called for the prudent use of antimicrobials in food animals to prevent AMR crisis (25, 26). The Indian subcontinent (India and Pakistan) is one of the largest dairy milk-producing regions in the world (12). However, to the best of our knowledge, there is no study on quantification of AMU at the farm level from the entire region of the Indian subcontinent.

A shift toward intensive livestock farming due to an increase in animal-based protein demand in LMICs has been positively linked with the excessive use of antimicrobials in food animals. In this study, we found an excessive amount of antimicrobial consumption in two of the corporate dairy farms, a growing industry in Pakistan. The total AMU of 66 mg/kg identified in this report is substantially higher than the global average of 45 mg/kg in cattle (4).

The number of on-farm treatments, quantified by the number of DDDA/1,000 cow-days, can be indicative of herd health status and the rationality of AMU. Indeed, a herd with poor health will receive more treatments and have higher ATI values than a herd with good health, and a herd with a good health status (i.e., low disease incidence rate) but relatively high ATI values suggests inappropriate metaphylactic or prophylactic AMU. ATI overestimation was checked by adjusting the weight of young stock to that of adult dairy cattle and by calculating per-day average DDDA for long-term preparations as reported previously (20, 23). However, antimicrobial overdosing or underdosing could not be accessed by ATI as no treatment records were maintained at farms and calculations were made using the labeled daily dose. In several cases, there was a discrepancy between the ATI and the AI. For example, unlike for parenteral treatments, because of low DDDA values, the ATIs of intramammary treatments were higher than the AIs (Table 2). Similarly, the observed number of ATIs for each AI in combination products was higher than that observed for single-ingredient products. Thereby, ATI is a measure of the number of treatments with reference to the number of cow-days, regardless of the amount of AI or DDDA used. A decimal ratio between the amount of AI used (AI) and the number of treatments per 1,000 cow-days (ATI), i.e., AI/ATI, reflects the relative value of the DDDA. A low AI/ATI ratio will indicate a low value for DDDA; in contrast, a high ratio will be suggestive of a high value for DDDA.

Total on-farm AMU (47.71 DDDA/1,000 cow-days and 65.88 mg/kg) was considerably higher than that reported from other countries, i.e., 4.2 DDDA/1,000 animal-days and 5.43 DDDA/cow/year or 14.88 DDDA/1,000 cow-days reported from Pennsylvania and Wisconsin, United States, respectively (15, 27); 14.35 DDDA/1,000 cow-days from Canada (28); 5.86 DDDA/cow/year or 16.05 DDDA/1,000 cow-days from Netherlands (23); 5.21 DDDA/LC/year or 14.27 DDDA/1,000 cow-days from Argentina (29); 20.78 DDDA/1,000 cow-days from Belgium (20); 1.27 PrDD_LU_/LU_pop−risk_/year, which would be equivalent to 3.48 DDDA/1,000 cow-days from Austria (30); 3.24 DDDvet/cow/year or 8.87 DDDA/1,000 cow-days from the United Kingdom (31); and 8.65 mg/kg from New Zealand (32).

We identified aminoglycoside and penicillin as the most commonly used antimicrobials in our study. In contrast, similar studies from European regions have shown penicillins and cephalosporins to be the most common antimicrobials used in the dairy sector (20, 23, 33–35). However, our results are in correspondence with studies from African LMICs where tetracyclines, aminoglycosides, and penicillins are the most highly used antimicrobials (36). The majority of antimicrobials were used for parenteral treatments followed by intramammary treatments, in line with the observations made in a Canadian study (28). Low ATI for intrauterine treatments is probably an underestimation as some of the parenteral antimicrobials might be used off-label for intrauterine therapies.

In this study, we observed a high intensity of AMU in the two corporate commercial dairy farms, with 91.4% of treatments consisting of critically and highly important antimicrobials for human medicine (Table 4). This finding highlights the fact that monitoring of AMU in commercial farming is crucial to the national efforts aiming to promote prudent use of antimicrobials and related stewardship programs (16).

The lack of internationally accepted standard methodology and of an abbreviation system for reporting AMU at the farm level hinders the quantification and comparison of data among different locations (17, 20, 29, 30, 32, 33, 37). However, the EMA has approved the number of DDDA/1,000 cow-days as a standard measure to report AMU in Europe (20). A system of high-resolution units quantifying AMU at the animal/herd-level where treatment data are available or contrariwise, along with their abbreviations, must be defined by OIE to streamline AMU data from different sources and regions of the world.

One of the main limitations of our study is that it was based on a convenient sample of farms that are not representative of the country's commercial dairy sector. Our results are therefore not generalizable to the rest of the Pakistani dairy sector. Another limitation is that, as the AMU calculations were based on inventory record data and the animal number was adjusted for a single dairy cow, AMU for each age group (calves, heifers, and cows) is not available, which may have led to the underestimation or overestimation of AMU in cows.

## Conclusion

This is the first attempt to calculate AMU in dairy animals in Pakistan. The total AMU was considerably higher when compared to that in international studies, with a large percentage of animal use of critically important antimicrobials for human medicine. Our baseline data will help policymakers to devise suitable antimicrobial stewardship programs for the emerging corporate commercial dairy sector in Pakistan.

## Data Availability Statement

All datasets presented in this study are included in the article/Supplementary Material.

## Author Contributions

MM: conceived and supervised the study. MM and MU: methodology. MN, RA, QA, JA, and MU: data collection and validation. MU and FF: data analysis and results interpretation. MM, MU, BA, MZ, and QA: manuscript writing and review. All authors contributed to the article and approved the submitted version.

## Conflict of Interest

The authors declare that the research was conducted in the absence of any commercial or financial relationships that could be construed as a potential conflict of interest.
