# Supplementary material for: First Case Report on Quantification of Antimicrobial Use in Corporate Dairy Farms in Pakistan
Source: Front Vet Sci. 2020 Nov 26;7:575848. doi: 10.3389/fvets.2020.575848 (PMC7725763; doi:10.3389/fvets.2020.575848)
Supplement: Supplementary file 1 [file Table_1.pdf]

Table S1. Active-ingredient (AI), antimicrobial treatment incidence (ATI) and milligrams of active ingredient used per kg of the total population weight (mg/PU) of different antimicrobials used at studied farms during the year 2018.

Sr #	Antimicrobial	Parenteral			Intramammary			Intrauterine			Oral			Total			
	**and Class**	**AI** [1]	**TF** [2]	**ATI** [3]	**AI kg**	**TF**	**ATI**	**AI kg**	**TF**	**ATI**	**AI kg**	**TF**	**ATI**	**AI kg**	**TF**	**ATI**	**mg/PU** [4]
	**Aminocoumarins**																
1	Novobiocin	-	-	-	0.17	0.00132	1.32	-	-	-	-	-	-	0.17	0.00132	1.32	0.08
	**Aminoglycosides**	31.55	0.00443	4.43	0.91	0.00557	5.57	0.01	0.00003	0.03	0.7	0.00102	1.02	33.17	0.01105	11.05	15.79
2	Dihydrostreptomycin	1.14	0.00017	0.17	0.17	0.00132	1.32	-	-	-	-	-	-	1.31	0.00148	1.48	0.62
3	Framycetin	-	-	-	0.09	0.00095	0.95	-	-	-	-	-	-	0.09	0.00095	0.95	0.04
4	Gentamicin	9.07	0.00334	3.34	0.1	0.00074	0.74	-	-	-	-	-	-	9.16	0.00408	4.08	4.36
5	Neomycin	-	-	-	0.56	0.00256	2.56	-	-	-	0.7	0.00102	1.02	1.26	0.00358	3.58	0.6
6	Streptomycin	21.34	0.00093	0.93	-	-	-	0.01	0.00003	0.03	-	-	-	21.35	0.00096	0.96	10.17
	**Aminopenicillins**	4.72	0.00073	0.73	0.18	0.00188	1.88	-	-	-	-	-	-	4.9	0.00261	2.61	2.33
7	Amoxicillin	4.72	0.00073	0.73	-	-	-	-	-	-	-	-	-	4.72	0.00073	0.73	2.25
8	Ampicillin	-	-	-	0.18	0.00188	1.88	-	-	-	-	-	-	0.18	0.00188	1.88	0.09
	**Aminopenicillins + β-lactam Inhibitors**																
9	Amoxicillin + Clavulanic acid	0.0088	0.0000013	0.0013	-	-	-	-	-	-	-	-	-	0.0088	0.0000013	0.0013	0.0042
	**Antifungals**																
10	Methylhydroxybenzoate	0.0009	0.00003	0.03	-	-	-	-	-	-	-	-	-	0.0009	0.00003	0.03	0.0004
	**Cephalosporins**	0.73	0.00096	0.96	1.52	0.0049	4.9	-	-	-	-	-	-	2.25	0.00585	5.85	1.07
11	Cefalonium (1st gen.)	-	-	-	1.37	0.00427	4.27	-	-	-	-	-	-	1.37	0.00427	4.27	0.65
12	Cephalexin (1st gen.)	-	-	-	0.15	0.0006	0.6	-	-	-	-	-	-	0.15	0.0006	0.6	0.07
13	Ceftiofur (3rd gen.)	0.47	0.00061	0.61	-	-	-	-	-	-	-	-	-	0.47	0.00061	0.61	0.22
14	Cefquinome (4th gen.)	0.26	0.00034	0.34	0.0018	0.00002	0.02	-	-	-	-	-	-	0.26	0.00036	0.36	0.13
	**Fluoroquinolones**	6.9	0.00372	3.72	-	-	-	-	-	-	-	-	-	6.9	0.00372	3.72	3.29
15	Enrofloxacin	5.95	0.00311	3.11	-	-	-	-	-	-	-	-	-	5.95	0.00311	3.11	2.83
16	Marbofloxacin	0.95	0.00062	0.62	-	-	-	-	-	-	-	-	-	0.95	0.00062	0.62	0.45
	**Macrolides**																
17	Tylosin	3.96	0.00147	1.47	-	-	-	-	-	-	-	-	-	3.96	0.00147	1.47	1.89
	**Penicillins**	8.11	0.003	3	0.84	0.00525	5.25	0.01	0.00003	0.03	-	-	-	8.96	0.00829	8.29	4.27
18	Cloxacillin	-	-	-	0.54	0.00204	2.04	-	-	-	-	-	-	0.54	0.00204	2.04	0.26
19	Penicillin G	8.11	0.003	3	0.3	0.00321	3.21	0.01	0.00003	0.03	-	-	-	8.42	0.00624	6.24	4.01
	**Phenicols**	11.75	0.00118	1.18	-	-	-	-	-	-	-	-	-	11.75	0.00118	1.18	5.6
20	Florfenicol	7.92	0.00063	0.63	-	-	-	-	-	-	-	-	-	7.92	0.00063	0.63	3.77
21	Thiamphenicol	3.83	0.00056	0.56	-	-	-	-	-	-	-	-	-	3.83	0.00056	0.56	1.82
	**Polymyxins**																
22	Colistin	0.65	0.00086	0.86	-	-	-	-	-	-	-	-	-	0.65	0.00086	0.86	0.31
	**Polypeptides**																
23	Bacitracin	-	-	-	0.04	0.00108	1.08	-	-	-	-	-	-	0.04	0.00108	1.08	0.02
	**Sulphonamides**	8.92	0.00008	0.08	-	-	-	0.35	0.00003	0.03	24.89	0.00203	2.03	34.16	0.00215	2.15	16.27
24	Sulphadiazine	-	-	-	-	-	-	-	-	-	13.83	0.00102	1.02	13.83	0.00102	1.02	6.58
25	Sulphadimidine	8.92	0.00008	0.08	-	-	-	-	-	-	11.06	0.00102	1.02	19.99	0.00109	1.09	9.52
26	Sulphathiazole	-	-	-	-	-	-	0.35	0.00003	0.03	-	-	-	0.35	0.00003	0.03	0.17
	**Tetracyclines**	31.15	0.00702	7.02	0.28	0.00108	1.08	-	-	-	-	-	-	31.42	0.0081	8.1	14.96
27	Oxytetracycline	31.15	0.00702	7.02	-	-	-	-	-	-	-	-	-	31.15	0.00702	7.02	14.83
28	Tetracycline	-	-	-	0.28	0.00108	1.08	-	-	-	-	-	-	0.28	0.00108	1.08	0.13
	**Total**	108.45	0.02349	23.49	3.93	0.02107	21.07	0.37	0.0001	0.1	25.59	0.00305	3.05	138.34	0.04771	47.71	65.88

[1] AI; the amount of active ingredient used in kg.

[2] TF; a ratio between the actual numbers of treatments and the maximum possible number of treatments and has no unit.

[3] ATI; the number of antimicrobial treatments per 1000 cow-days in DDDA/1,000 cow-days.

[4] mg/PU; milligrams of active ingredient used per kilogram of total animal biomass in mg/kg.
